# Lipid Metabolic Versatility in *Malassezia* spp. Yeasts Studied through Metabolic Modeling

**DOI:** 10.3389/fmicb.2017.01772

**Published:** 2017-09-14

**Authors:** Sergio Triana, Hans de Cock, Robin A. Ohm, Giovanna Danies, Han A. B. Wösten, Silvia Restrepo, Andrés F. González Barrios, Adriana Celis

**Affiliations:** ^1^Department of Biological Sciences, Universidad de los Andes Bogotá, Colombia; ^2^Grupo de Diseño de Productos y Procesos, Department of Chemical Engineering, Universidad de los Andes Bogotá, Colombia; ^3^Structural and Computational Biology Unit, European Molecular Biology Laboratory Heidelberg, Germany; ^4^Microbiology, Department of Biology, Faculty of Science, Utrecht University Utrecht, Netherlands

**Keywords:** *Malassezia*, genome, metabolic reconstruction, FBA, lipid metabolism

## Abstract

*Malassezia* species are lipophilic and lipid-dependent yeasts belonging to the human and animal microbiota. Typically, they are isolated from regions rich in sebaceous glands. They have been associated with dermatological diseases such as seborrheic dermatitis, pityriasis versicolor, atopic dermatitis, and folliculitis. The genomes of *Malassezia globosa*, *Malassezia sympodialis*, and *Malassezia pachydermatis* lack the genes related to fatty acid synthesis. Here, the lipid-synthesis pathways of these species, as well as of *Malassezia furfur*, and of an atypical *M. furfur* variant were reconstructed using genome data and Constraints Based Reconstruction and Analysis. To this end, the genomes of *M. furfur* CBS 1878 and the atypical *M. furfur* 4DS were sequenced and annotated. The resulting Enzyme Commission numbers and predicted reactions were similar to the other *Malassezia* strains despite the differences in their genome size. Proteomic profiling was utilized to validate flux distributions. Flux differences were observed in the production of steroids in *M. furfur* and in the metabolism of butanoate in *M. pachydermatis*. The predictions obtained via these metabolic reconstructions also suggested defects in the assimilation of palmitic acid in *M. globosa*, *M. sympodialis*, *M. pachydermatis*, and the atypical variant of *M. furfur*, but not in *M. furfur.* These predictions were validated via physiological characterization, showing the predictive power of metabolic network reconstructions to provide new clues about the metabolic versatility of *Malassezia*.

## Introduction

*Malassezia* species are lipophilic and lipid-dependent yeasts that are frequently encountered in the human and animal microbiota. Usually, they are isolated from regions rich in sebaceous glands. They have been associated with dermatological diseases such as seborrheic dermatitis (SD), pityriasis versicolor, atopic dermatitis, and folliculitis (Boekhout, 2010). The increasing number of *Malassezia* isolations from systemic infections shows that members of this genus are emerging opportunistic agents (Gaitanis et al., 2012; Arendrup et al., 2014).

The lipid dependency of the *Malassezia* species has been confirmed through the genome sequences of 14 species within the genus (Xu et al., 2007; Gioti et al., 2013; Triana et al., 2015; Wu et al., 2015). The genomes lack the cytosolic fatty acid synthase (FAS) gene, thus explaining why they cannot synthesize the fatty acid palmitate *de novo*. On the other hand, lipase, phospholipase, and sphingomyelinase genes that are involved in the release of fatty acids from the host are present, enabling lipid synthesis in *Malassezia* species (Xu et al., 2007). Genes homologous to FAS genes have also been identified, but they have been predicted to have different functions (Xu et al., 2007; Triana et al., 2015).

The *Malassezia* genus shows different lipid-assimilation phenotypes as well as differences in the number of lipids that are required in the growth medium. *Malassezia furfur* usually assimilates different kinds of Tween including Tween 20, 40, 60, and 80. However, atypical strains have been identified, including strains that can only assimilate Tween 80 (Gonzalez et al., 2009), and strains that are reported to be lipid-independent (Zinkeviciene et al., 2012). In the latter case, lipid-independent growth was only tested in Sabouraud glucose agar medium, which still contains lipids (Wu et al., 2015). *Malassezia pachydermatis* was also believed to be lipid-independent (Juntachai et al., 2009). However, recent findings show that this species is actually a more versatile lipid-dependent yeast also lacking a FAS gene (Triana et al., 2015; Wu et al., 2015). The *Malassezia* assimilation assay is widely used to determine the lipid requirement of strains (Boekhout, 2010). For instance, growth on Tween is indicative of a role being played by external lipase(s) that release the fatty acid tail from the non-ionic detergent. Analysis of the genomes of *Malassezia* spp. revealed a collection of genes encoding lipases, phospholipases, and sphingomyelinases (Xu et al., 2007; Gioti et al., 2013; Triana et al., 2015; Wu et al., 2015) that are most likely involved in the release of fatty acids from a variety of lipid compounds, for example, such as those produced by the sebaceous glands in the skin of the host. Uptake of these fatty acids and their subsequent use in various lipid-biosynthesis routes is required to sustain the growth of *Malassezia* species.

Genomic data and Constraints Based Reconstruction and Analysis (COBRA)-based models (Murabito et al., 2009) can be used to reconstruct the cellular metabolism of an organism in a mathematical model. Such networks can be used to predict a cell’s behavior under different conditions or disorders (Orth et al., 2014). Flux balance analysis (FBA) models are constraint-based approaches suitable for studying the range of possible phenotypes of a metabolic system (Murabito et al., 2009).

The aim of this work was to investigate the genomic and metabolic differences between reference strains of four *Malassezia* species and an atypical *M. furfur* strain. To this end, we used metabolic modeling, using genomics and proteomics data. The results show flux differences in the production of steroid in *M. furfur* and the metabolism of butanoate in *M. pachydermatis*. In addition, defects in the assimilation of palmitic acid in *Malassezia globosa*, *Malassezia sympodialis*, *M. pachydermatis*, and the atypical variant of *M. furfur* (4DS), were suggested, but not for *M. furfur* CBS 1878. These predictions were validated via culturing on defined media.

## Materials and Methods

### Strains and Growth Conditions

The reference *Malassezia* strains *M. globosa* CBS 7986, *M. sympodialis* CBS 7222, *M. pachydermatis* CBS 1879, and *M. furfur* CBS 1878 purchased from Fungal Biodiversity Center (Westerdijk Institute, Utrecht, Netherlands), as well as a previously reported isolate of *M. furfur* with atypical assimilation of Tween 80 (4DS) (from now on referred to as atypical *M. furfur*) (Gonzalez et al., 2009) were used in this study and recovered in modified Dixon agar (mDixon): 36 g L^-1^ mycosel agar (BD, United States), 20 g L^-1^ Ox-bile (Sigma–Aldrich, United States), 36 g L^-1^ malt extract (Oxoid, UK), 2 mL L^-1^ glycerol (Sigma–Aldrich, United States), 2 mL L^-1^ oleic acid (Sigma–Aldrich, United States), and 10 mL L^-1^ Tween 40 (Sigma–Aldrich, United States) for 4–5 days at 33°C (Boekhout, 2010). The genomes of *M. globosa* (Xu et al., 2007), *M. pachydermatis* (Triana et al., 2015), and *M. sympodialis* (Gioti et al., 2013) have been sequenced and are available in GenBank under the accession numbers GCA_000181695.1, GCA_001278385.1, and GCA_000349305.2. For the phylogenetic analysis the *Malassezia* genomes reported by Wu et al. (2015) and the *Ustilago maydis* genome (Kämper et al., 2006) were used, these are available in GenBank under BioProject ID: PRJNA286710 and accession number GCA_000328475.2, respectively.

### DNA Extraction

The *M. furfur* strains that were sequenced in this study were recovered in modified mDixon for 4–5 days at 33°C (Boekhout, 2010). Genomic DNA was extracted as described (Gonzalez et al., 2009).

### Sequencing and Genome Assembly

DNA from the *M. furfur* strains was sequenced at the Beijing Genomics Institute (Shenzhen) using the Illumina HiSeq 2000 platform. Two runs of 100-bp paired-end reads and 200-bp insert-size libraries were undertaken following standard Illumina protocols. The quality of the reads was analyzed using FastQC software (Andrews, 2010) and trimmed and filtered based on quality using Flexbar (Dodt et al., 2012). *De novo* assembly was performed using the CLC Assembly Cell software (CLC bio, 2014) using default parameters. The resulting contigs were scaffolded using SSPACE_Basic script (Boetzer et al., 2011), discarding scaffolds <1,000 bp. Gaps in the scaffolds were filled with the GapFiller script (Boetzer and Pirovano, 2012). Assembly statistics such as N50, N75, L50, L75, and GC content, were computed with the QUAST software (Gurevich et al., 2013). The average genome coverage was calculate by mapping the reads to the assembly with Bowtie (Langmead et al., 2009) using default parameters and calculating the coverage per base using BEDTools (Quinlan and Hall, 2010). This whole-genome shotgun project has been deposited at DDBJ/EMBL/GenBank under the accession numbers MATP00000000 and LMYL00000000. The version described herein are LMYL01000000 and MATP01000000.

### Assembly Comparisons

To determine the genome similarity among the species and to identify the presence of repetitions in the genomic and protein level, we did the following. The assemblies of the *M. furfur* strains were aligned with each other and with the genomes of *M. globosa* (Xu et al., 2007), *M. sympodialis* (Gioti et al., 2013), and *M. pachydermatis* (Triana et al., 2015) using Nucmer (a maximum gap between two adjacent matches in a cluster of 90 bp and a minimum length of a maximal exact match of 20 bp) and Promer (a maximum gap between two adjacent matches in a cluster of 30 amino acids and a minimum length of a maximal exact match of six amino acids), which evaluates the six-frame translation of the nucleotide sequence. Mummer (Delcher et al., 2002) alignments were plotted and the genome coverage per nucleotide was calculated with the BEDTools suite Coverage tool (Quinlan and Hall, 2010). The percentage of matches in each pairwise comparison was computed using custom python scripts.

### Annotation

The assemblies of the *M. furfur* strains were annotated using the MAKER 2 pipeline (Cantarel et al., 2007). To this end, a set of 109,264 previously reported Ustilaginomycotina proteins from NCBI protein database was used. In addition, genes predicted by CEGMA (Parra et al., 2007) and GeneMark (Borodovsky and Lomsadze, 2011), 1,413 expressed sequence tags (ESTs) from *Malassezia* spp., and results from the *ab initio* gene predictors SNAP (Korf, 2004) and Augustus (Stanke et al., 2004) were used as genetic evidence for the annotation. MAKER was run two consecutive times. The first run included proteins, ESTs, and predicted genes to identify genes within the scaffolds. The first output obtained from MAKER was converted into a model for SNAP and a training set for Augustus. Subsequently, the *ab initio* results were provided as an input model for the second MAKER run. The statistics of the resulting annotation were calculated with genome tools (Gremme et al., 2013).

Functional annotation of the predicted genes was performed using Blast2GO (Conesa and Götz, 2008), which included BlastX (Altschul et al., 1990) and an InterProScan annotation (Quevillon et al., 2005). To determine the number of duplicated proteins in the *M. furfur* genomes, CD-Hit (Li and Godzik, 2006) was run with an identity threshold of 90%. Non-coding repeated sequences within the genomes were analyzed using RepeatMasker^1^ by running them against the RepBase library.

To assess whether genes involved in the formation of free fatty acid precursors are absent in the *M. furfur* genomes, as is the case for *M. globosa*, *M. sympodialis*, and *M. pachydermatis*, 2,382 fungal and bacterial genes encoding FAS and 954 polyketide synthase (PKS) genes were compared with the predicted genes and proteins of the genomes using blastp, blastn, tblastn from the blast suite version 2.2.29 (Altschul et al., 1990) (parameters by default) and phmmer and hmmsearch from the HMMER software, version 3.1b2^2^. Further validation of missing InterProScan domain was manually done by comparing the missing domain sequence from the *Malassezia* species presenting the domain with the predicted protein of the other species.

### Phylogenetic Analysis

To estimate the phylogeny of the available *Malassezia* species (Xu et al., 2007; Gioti et al., 2013; Triana et al., 2015; Wu et al., 2015), genes were predicted on each genome assembly using Augustus 3.0.2 (Stanke et al., 2004) with the parameters that were previously optimized for *M. furfur* CBS 1878. *U. maydis* was used as an outgroup. Highly conserved genes were identified using BUSCO 2.0 (Simão et al., 2015) using the gene set “fungi odb9.” These sequences of each species were concatenated, aligned using MAFFT version 7.309 (Katoh and Standley, 2013), and the well-aligned regions were extracted using Gblocks 0.91b (Castresana, 2000). This resulted in 62,988 amino acid positions. FastTree 2.1.9 (Price et al., 2010) was used to reconstruct the species tree.

### Metabolic Network Reconstruction

The predicted proteins were compared to the KEGG database (Kanehisa and Goto, 2000) using the KAAS server (Moriya et al., 2007) and Blast2GO (Conesa and Götz, 2008) to obtain corresponding Enzyme Commission (EC) numbers. These numbers were used to retrieve the associated reactions from KEGG and to map the corresponding metabolic pathways. The directionality of each reaction was determined using the literature, the MetaCyc database (Caspi et al., 2014), and the Gibbs free energy obtained with the group contribution method (Jankowski et al., 2008). The reaction nomenclature was converted to the Metanetx identifiers in order to have a more cohesive nomenclature in the network (Ganter et al., 2013). Furthermore, the metabolic core was determined as the reactions shared among the five strains.

### Compartmentalization and Curation

The predicted enzymes were analyzed with the subCELlular LOcalization predictor (CELLO; Yu et al., 2004), a peptide localization predictor that uses support vector machines based on n-peptide compositions. The significant compartment was selected and added to the reactions of the enzyme. Transport reactions were added to the network according to the genome annotation and the literature review. The metabolites with production and consumption problems were identified and missing data were imputed using an iterative approach with the GapFind and GapFill algorithms (Boetzer and Pirovano, 2012) implemented in the General Algebraic Modeling System (GAMS) (Bruce, 2013) using the minimal media defined for iMM904 *Saccharomyces cerevisiae* (Zomorrodi and Maranas, 2010) supplemented with oleic acid and glucose as the sole carbon source.

In addition to a manual curation based on the literature and visualization in Cytoscape (Shannon et al., 2003), an in-house exchange-reaction database and the complete metabolic reactions’ repository for Metanetx (Ganter et al., 2013) were used to detect unconnected metabolites, and to calculate the topological statistics of the network.

### Flux Balance Analysis

A stoichiometric matrix (*S*) was obtained from the metabolic network using in-house Perl scripts to obtain a system of linear equations, *S* × *v* = 0, where *v* is the flux vector. System constraints included the lower and upper bounds of reaction fluxes. The system allowed 0.000–1,000 mmol gDW^-1^ h^-1^ for irreversible reactions and -1,000 to 1,000 mmol gDW^-1^ h^-1^ for reversible reactions. The system was solved to identify the theoretical limits for different fluxes in the metabolic system using GAMS (GAMS Development Corp., Washington, DC, United States) software. The linear programming presented here was developed with solver CPLEX 12.6.0.0 with an optimization tolerance of 10^-6^. A modified biomass production reaction (Supplementary Table S1) of iMM904 *S. cerevisiae* as an objective function (Zomorrodi and Maranas, 2010) were fixed as parameters for the optimization process. Furthermore the same media used in the GapFill process, the minimal media defined for iMM904 *S. cerevisiae* (Zomorrodi and Maranas, 2010) supplemented with oleic acid and glucose (uptake rate of 10 mmol gDW^-1^ h^-1^) in anaerobic conditions (oxygen uptake rate of 2 mmol gDW^-1^h^-1^) was used for the simulation. This optimization problem aimed to solve the maximization of the flux through the reaction of biomass used as objective functions. The resulting flux distributions were filtered with a cutoff of ±0.05 mmol gDW^-1^ h^-1^ to allow a better visualization of the reactions that can carry fluxes and plotted as a heatmap in R (R Core Team, 2013).

### Proteomic Profiling

Protein extraction was carried out as described (Kim et al., 2010). Strains were grown at 180 rpm and 33°C on DB (20 g L^-1^ Ox-bile, Sigma–Aldrich, United States), 36 g L^-1^ malt extract (Oxoid, UK), 6 g L^-1^ peptone (Oxoid, UK), 2 mL L^-1^ glycerol (Sigma–Aldrich, United States), 2 mL L^-1^ oleic acid (Sigma–Aldrich, United States), 10 mL L^-1^ Tween 40 (Sigma–Aldrich, United States), and 500 mg L^-1^ chloramphenicol (Sigma–Aldrich, United States). Aliquots of 5 mL were taken in the early exponential and early stationary phase and centrifuged at 26,000 *g* for 10 min. The resulting pellet was washed three times with PBS, after which it was resuspended in extraction buffer [1:10 cell to extraction buffer v/v ratio; 125 mM ammonium bicarbonate (Sigma–Aldrich, United States), 20 mM 𝜀-aminocaproic acid (Sigma–Aldrich, United States), 5 mM ethylenediaminetetraacetic acid (Sigma–Aldrich, United States), and 1 mM phenylmethylsulfonyl fluoride (Thermo Fisher Scientific, United States)]. Cells were disrupted by vortexing for 10 min with 4-mm silica beads and centrifuged at 26,000 *g* and 4°C for 10 min. Proteins in the supernatant were precipitated with 20% TCA (Sigma–Aldrich, United States) (Wood, 1991). The amount of protein was quantified using a Nanodrop (Thermo Fisher Scientific, United States) and visualized by SDS-PAGE electrophoresis. The protein extraction was carried out five times per sample.

The extracted proteins were sent to the proteomic center of the University of California at Davis^3^. Protein profiling for each sample was carried out using the mass spectrometer Michrom HPLC Paradigm type, the Q-mass spectrometer ionization Proxeon Exactive nano-spray, and the Easy-LC II HPLC.

The identification and annotation of the proteins was performed using Scaffold (Proteome Software Inc., Portland, OR, United States) and proteins were compared against the Uniprot Ustilaginomycotina proteins and the *Malassezia* spp. predicted proteins. The mass spectrometry proteomics data have been deposited in the ProteomeXchange Consortium via the PRIDE (Vizcaíno et al., 2016) partner repository with the dataset identifier PXD004523. The resulting proteins were clustered with CD-Hit to compare the proteins among the samples. These clusters were analyzed statistically with R using the normalized total spectra count (R Core Team, 2013). To validate the network, proteins related to the reactions in the genomic model were selected (excluding those metabolic and transport reactions added with GapFill that did not have an associated enzyme) and compared to the abundance of the proteins in each extract using at least two replicates with 95% identity.

### Physiological Characterization of Lipid Assimilation

To determine the growth on different Tween varieties and with fatty acids, the strains were first grown on mDixon at 33°C for 7 days. The fungal cells were suspended in 3 ml of water with 0.1% Tween 80 used to inoculate 27 mL of minimal medium (MM) [containing per liter: 10 mL K-buffer pH 7.0 (200 g L^-1^ K_2_HPO_4_; Sigma–Aldrich, United States), 145 g L^-1^ KH_2_PO_4_ (Sigma–Aldrich, United States), 20 mL M-N (30 g L^-1^ MgSO_4_.7H_2_O; Sigma–Aldrich, United States), 15 g L^-1^ NaCl (Sigma–Aldrich, United States), 1 mL 1% CaCl_2_.2H_2_O (Sigma–Aldrich, United States) (w/v), 10 mL 20% glucose (Sigma–Aldrich, United States) (w/v), 10 mL 0.01% FeSO_4_ (Sigma–Aldrich, United States) (w/v), 5 mL spore elements (100 mg L^-1^ ZnSO_4_.7H_2_O; Sigma–Aldrich, United States), 100 mg L^-1^ CuSO_4_.5H_2_O (Sigma–Aldrich, United States), 100 mg L^-1^ H_3_BO_3_ (Sigma–Aldrich, United States), 100 mg L^-1^ MnSO_4_.H_2_O (Sigma–Aldrich, United States), l00 mg L^-1^ Na_2_MoO_4_.2H_2_O (Sigma–Aldrich, United States), and 2.5 mL 20% NH_4_NO_3_ (Sigma–Aldrich, United States) (w/v)] containing 4 mM Tween [20, 40, 60 or 80 (Sigma–Aldrich, United States)], or 4 mM oleic acid (Carlo Erba), or palmitic acid (Merck) supplemented with 1% Brij-58 (Sigma–Aldrich, United States), an emulsifier that is not metabolized. It did not support the growth of the FAS mutant of the yeast *S. cerevisiae* (Schweizer and Bolling, 1970) that was grown for 3 days at 33°C. Subsequently, 0.3 mL was used to inoculate 29.7 mL of fresh MM containing either 4 mM Tween 20, 40, 60, 80, oleic acid, and/or palmitic acid in 1% Brij-58, with mDixon broth as the positive control. Growth was followed during 8 days in the medium containing Tween 40, palmitic acid, oleic acid, or mixtures of palmitic and oleic acid by determining the colony-forming unit (CFU) by plating on mDixon plates with subsequent incubation at 33°C. In the medium containing Tweens and oleic acid, the optical density (OD) at 600 nm was measured and plating aliquots of the liquid cultures on mDixon plates at 33°C was used to determine the viability of the cells after 8 days of growth. The fatty acids used for culturing were analyzed for composition via a gas chromatography–flame ionization detector; separation was reached using an RTX-Wax column (30 m × 0.25 mm × 0.5 μm) of RESTEK^®^. FAMEs were identified by comparing their retention times with those identified with a Supelco^®^ 37 Component FAME Mix standard. Quantification was intended as a relative concentration. Palmitic acid (Merck) contained 98% palmitic acid and 2% elaidic acid, an unsaturated acid. The oleic acid (Carlo Erba) contained 78% oleic acid and, in addition, we detected polyunsaturated fatty acids (10% of linoleic acid), unsaturated fatty acids (3% palmitoleic acid and 2% elaidic acid), and saturated fatty acids (6% of palmitic acid and 1% heptadecanoic acid).

## Results

### Genome Assembly and Pairwise Comparisons

The draft genomes of *M. furfur* CBS 1878 and the atypical *M. furfur* 4DS were assembled from a shotgun Illumina HiSeq 2000-Paired data set using the CLC-assembler (CLC bio, 2014). The assemblies yielded 2,084 scaffolds (N50 = 23 kb) and 3,577 scaffolds (N50 = 42 kb), respectively, corresponding to nuclear genomes of 14.19 and 10.38 Mb. The summary of the genome assembly statistics calculated by QUAST (Gurevich et al., 2013) is shown in **Table 1**.

**Table 1:** Assembly statistics calculated by QUAST (Gurevich et al., 2013) and BEDtools (Quinlan and Hall, 2010).

	*Malassezia*	Atypical
Assembly	*furfur*	*Malassezia furfur*
Number of contigs (≥0 bp)	6,968	17,882
Number of contigs (≥1,000 bp)	1,249	877
Total length (≥0 bp)	15,780,944	14,951,138
Total length (≥1,000 bp)	13,644,665	8,614,553
# Scaffolds	2,084	3,577
Largest scaffold	110,895	562,614
Total length	14,194,927	10,380,899
GC (%)	63.95	63.01
N50	23,366	42,453
N75	10,269	2,449
L50	171	50
L75	400	295
Number of Ns in the assembly (per 100 kb)	41.75	127.69
Average coverage	605×	861×

*The genome assembly was performed using the CLC-assembler (CLC bio, 2014) and scaffolding was carried out using Sspace (Boetzer et al., 2011) and Gapfiller (Boetzer and Pirovano, 2012).*

A pairwise Nucmer comparison at the nucleotide level showed that *M. furfur* and the atypical *M. furfur* had more repetitions and/or duplications in their genomes than *M. globosa*, *M. pachydermatis*, and *M. sympodialis* did (Supplementary Figure S1 and **Table 2**), with 30.8 and 7% of the genomes of *M. furfur* and of the atypical *M. furfur* strains, respectively, representing multiple matches or repetitions. In contrast, less than 1% of the genomes of *M. globosa*, *M. pachydermatis*, and *M. sympodialis* corresponded to multiple matches.

**Table 2:** Percentage of the genome with zero (0), one (1), or multiple (>1) matches in the Nucmer genome alignment (Boetzer and Pirovano, 2012) with a maximum gap between two adjacent matches in a cluster of 90 bp and a minimum length of a maximal exact match of 20 bp.

	Atypical *M. furfur*			*M. furfur*			*M. globosa*			*M. pachydermatis*			*M. sympodialis*		
Nucmer	0	1	>1	0	1	>1	0	1	>1	0	1	*>1*	0	1	>1
Atypical *M. furfur*	0.1%	92.9%	7.0%	3.9%	43.2%	52.8%	98.1%	1.9%	0.0%	96.6%	3.3%	0.0%	94.6%	5.4%	0.0%
*M. furfur*	12.6%	75.1%	12.3%	0.0%	69.1%	30.8%	96.4%	3.6%	0.1%	95.1%	4.9%	0.0%	93.2%	6.7%	0.0%
*M. globosa*	99.2%	0.8%	0.0%	99.2%	0.6%	0.2%	0.0%	99.7%	0.3%	98.8%	1.2%	0.0%	98.7%	1.3%	0.0%
*M. pachydermatis*	97.5%	2.5%	0.0%	97.5%	1.6%	1.0%	98.7%	1.3%	0.0%	0.0%	99.8%	0.2%	96.6%	3.4%	0.0%
*M. sympodialis*	95.6%	4.4%	0.0%	94.8%	3.1%	2.1%	98.4%	1.5%	0.0%	96.4%	3.6%	0.0%	0.2%	98.9%	0.9%

The Nucmer alignment showed significant sequence divergence between the four species. The biggest difference was observed for *M. globosa*, which only showed 0.8% identity using exact matches of at least 20 bp when compared to *M. furfur* or the atypical *M. furfur* genomes. This was <1.2% when the *M. globosa* genome was compared to *M. pachydermatis* and *M. sympodialis*. As expected, the most similar strains were *M. furfur* and the atypical *M. furfur* strains with a similarity >80% (**Table 2**). A higher degree of similarity was observed when evaluating the six translation frames using Promer (Supplementary Figure S2 and **Table 3**). The biggest differences were found when *M. furfur* and *M. sympodialis* were compared, with 70.7% zero matches. The second highest difference was between *M. globosa* and *M. furfur*, with approximately 48% of zero matches. As expected, the highest similarity was observed in the case of the *M. furfur* strains with only 3.4% of zero matches.

**Table 3:** Percentage of the genome with zero (0), or one or more (1) matches in the Promer genome alignment (Delcher et al., 2002) with a maximum gap between two adjacent matches in a cluster of 30 amino acids and a minimum length of a maximal exact match of six amino acids.

	Atypical *M. furfur*		*M. furfur*		*M. globosa*		*M. pachydermatis*		*M. sympodialis*	
Promer	0	1	0	1	0	1	0	1	0	1
Atypical *M. furfur*	0.10%	99.90%	3.40%	96.60%	49.60%	50.40%	44.50%	55.50%	44.50%	55.50%
*M. furfur*	11.80%	88.20%	0.00%	100.00%	76.70%	23.30%	73.10%	26.90%	70.70%	29.30%
*M. globosa*	49.50%	50.50%	48.60%	51.40%	0.00%	100.00%	38.50%	61.50%	39.60%	60.40%
*M. pachydermatis*	40.30%	59.70%	39.20%	60.80%	33.60%	66.40%	0.00%	100.00%	28.40%	71.60%
*M. sympodialis*	37.50%	62.50%	35.70%	64.30%	31.80%	68.20%	26.00%	74.00%	0.20%	99.80%

**FIGURE 1:**
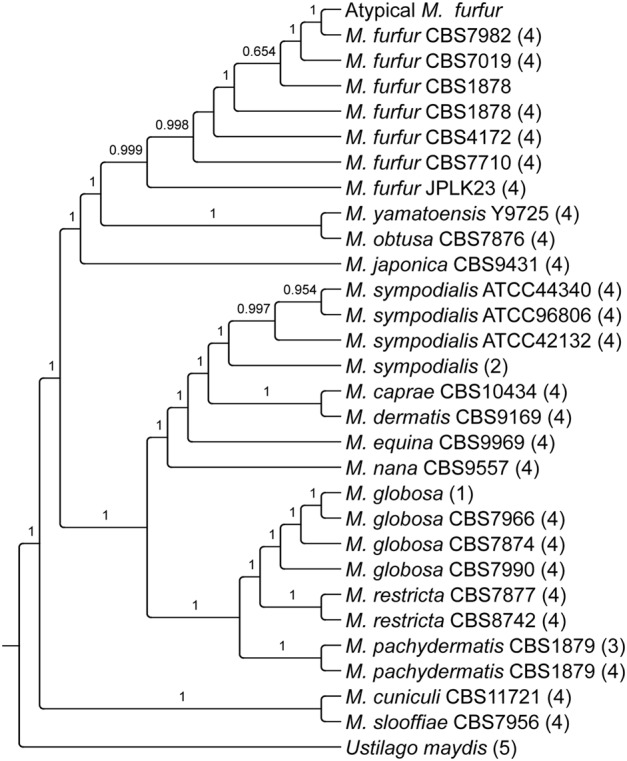
Phylogenetic analysis of the *Malassezia* genus reconstructed from highly conserved genes identified with BUSCO 2.0 (Simão et al., 2015). (1) Xu et al., 2007, (2) Gioti et al., 2013, (3) Triana et al., 2015, (4) Wu et al., 2015, (5) Kämper et al., 2006.

To establish the taxonomic position of the two reported *M. furfur* sequences in this study with the already published *Malassezia* genomes, we built a phylogenetic tree using highly conserved genes (**Figure 1**). This phylogeny showed that atypical *M. furfur* clustered with *M. furfur* CBS 7982 and our sequence of *M. furfur* CBS 1878 clustered with the previously reported sequence of this strain.

### Assembly Annotation

**FIGURE 2:**
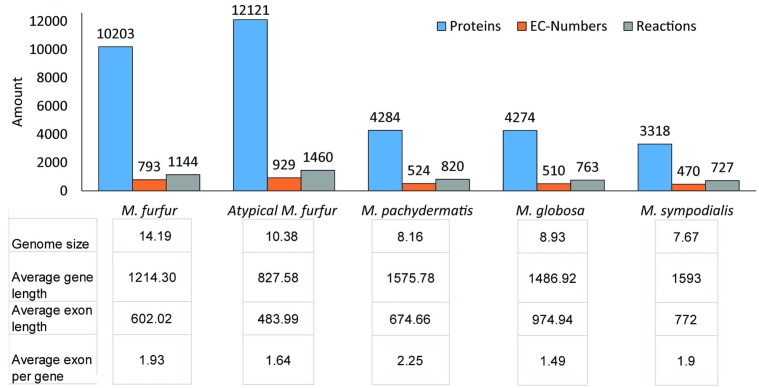
Annotation data for each species: for the *M. furfur* strains, the predicted proteins were obtained from MAKER (Cantarel et al., 2007). For all the strains, the ECs were obtained from KAAS (KEGG Automatic Annotation Server) (Moriya et al., 2007) and Blast2GO (Conesa and Götz, 2008), the reactions were retrieved from the KEGG database, and the annotation statistics were calculated with genome tools (Gremme et al., 2013).

A total of 10,203 and 12,131 protein-encoding genes were predicted for *M. furfur* and the atypical *M. furfur* strain using multiple lines of evidence (*ab initio* predictors, ESTs from *M. globosa*, and protein alignments). Functional annotation showed that both *M. furfur* strains contained twice the amount of proteins when compared to the other *Malassezia* species. Yet, the number of EC numbers and reactions were similar, as can be seen in **Figure 2**. The average genome length, exon length, and the number of exons per gene for the five genomes analyzed are presented in **Figure 2**.

The genomes of *M. furfur* and the atypical *M. furfur* contained 7,570 and 10,434 protein clusters, respectively, as predicted with CD-Hit (Li and Godzik, 2006). The total number of non-coding repetitions found using RepeatMasker was approximately 1.61 and 1.32% for *M. furfur* and the atypical *M. furfur* strain, respectively. Most repetitions were found to be low-complexity repeats and long terminal repeat elements.

**FIGURE 3:**
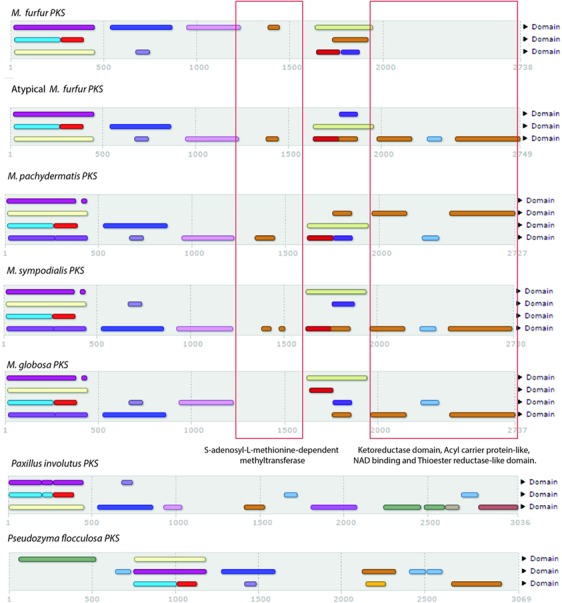
Domains obtained with InterProScan (Quevillon et al., 2005) of the five polyketide synthases (PKSs) of *Malassezia* species compared with *Paxillus involutus* and *Pseudozyma flocculosa*.

To search for genes related to lipid biosynthesis, a total of 2,382 FASs (i.e., 179 fungal and 2,203 bacterial) were compared with the predicted proteins of the *M. furfur* strains using blastp, blastn, and tblastn (Altschul et al., 1990) and phmmer and hmmsearch (see text footnote 2). No FAS genes were identified in the two genomes, but several PKS genes (three in *M. furfur* and one in atypical *M. furfur*) were found (**Figure 3**). The PKSs from the five species shared most of the domains, with the exception of *M. globosa*, which lacked the *S*-adenosyl-L-methionine-dependent methyltransferase domain, and *M. furfur*, which lacked the ketoreductase domain, acyl carrier protein-like domain, NAD-binding domain, and the thioester reductase-like domain in its three predicted PKSs. Out of 954 fungal homologs, the PKS of the basidiomycete *Paxillus involutus* was the most similar to that of the atypical *M. furfur*, with an identity of 31%. The most similar PKSs to those of *Malassezia* were found in basidiomycetes. The closest homolog of the PKS of *M. furfur* was from *Hydnomerulius pinastri*, with an identity of 29.8%, while an *Auricularia delicata* homolog was most similar to the PKS of *M. pachydermatis* and *M. sympodialis*, showing 29.9 and 31% similarity, respectively. In the case of *M. globosa*, the most similar PKS was that of *Coniophora puteana*, with an identity of 26.8%.

**FIGURE 4:**
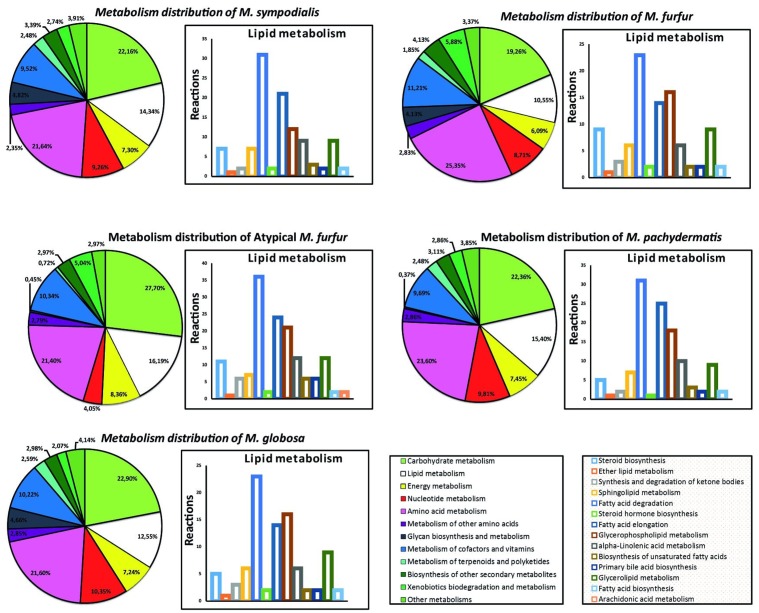
Metabolism distribution predicted from each species and an expanded distribution for the lipid metabolism.

The distribution of the genes in the genomes of the five *Malassezia* strains was similar when analyzing 13 categories of metabolic genes (**Figure 4**). The metabolic core (Supplementary Figure S3) represented 628 reactions that were shared between the strains. These were mainly distributed in the carbohydrate and amino acid metabolism, and lipid metabolism. When examining the lipid metabolism in more detail, arachidonic acid biosynthesis was only present in the atypical *M. furfur*. Differences in the fungal steroid biosynthesis were also found. Around 10 reactions involved in this pathway were present in the *M. furfur* strains, which was higher than those in the other species with less than six reactions. Similarly, 36 reactions associated with fatty acid degradation were detected in atypical *M. furfur*, a higher number than that found in the other species studied (23–31 reactions) (**Figure 4**).

### Network Construction and Curation

**FIGURE 5:**
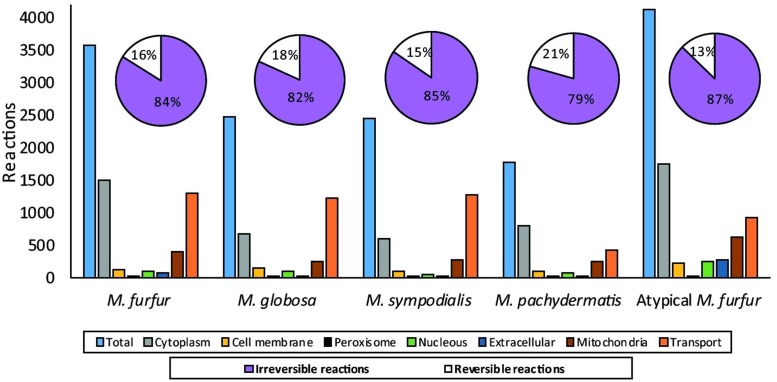
Distribution of the reactions after compartmentalization with the subCELlular LOcalization predictor (CELLO) (Yu et al., 2004) and the percentage of reversible and irreversible reactions determined from the Gibbs free energy of the reactions calculated with the group contribution approach and from the literature.

**FIGURE 6:**
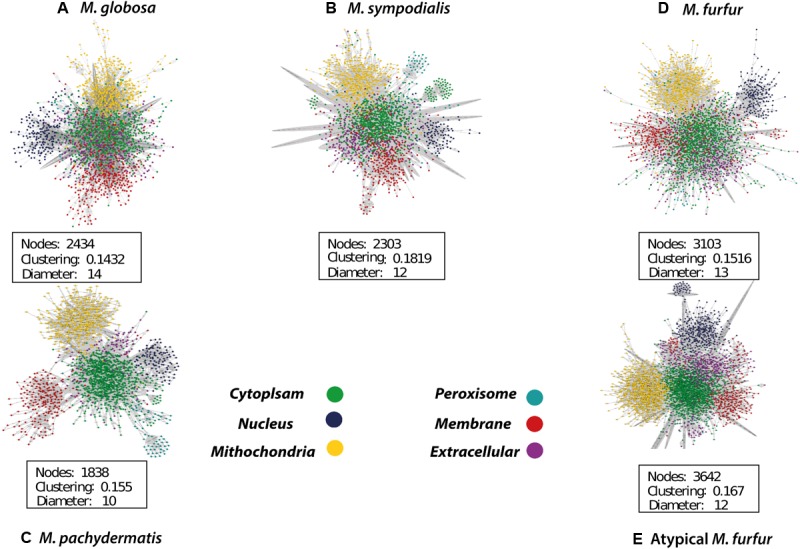
Compartmentalized metabolic network of *Malassezia globosa* **(A)**, *Malassezia sympodialis* **(B)**, *Malassezia pachydermatis* **(C)**, *Malassezia furfur* **(D)**, and atypical *Malassezia furfur* **(E)** visualized in Cytoscape (Shannon et al., 2003).

The database search and the group contribution method used to calculate the Gibbs free energy predicted that 15–26% of the reactions of the five *Malassezia* strains were irreversible. Compartmentalization analyses showed a similar distribution of reactions among the species. However, differences were observed in the number of reactions when the *M. furfur* strains were compared to the other species. This corresponded to cytoplasmic and mitochondrial reactions (**Figure 5**). After the gap-filling approach to reduce the network pathologies, around 250 reactions were added to *M. globosa* and *M. sympodialis*, 300 to *M. pachydermatis*, 600 to *M. furfur*, and 660 to the atypical *M. furfur* strain. The final networks ended up with 2,162 metabolites in *M. globosa*, 2,303 in *M. sympodialis*, 1,838 in *M. pachydermatis*, 3,103 in *M. furfur*, and 3,642 in the atypical *M. furfur* strain. The final metabolic networks (Supplementary Data Sheet 2) were visualized in Cytoscape using the compartment as a discrete mapping category to color the nodes. A topological analysis was performed (**Figure 6**). The clustering coefficients were around 0.14 and 0.18 and the diameter of the networks ranged between 10 and 15 nodes.

### Flux Balance Analysis

FBA was carried out and the resulting predicted biomass fluxes that were used as objective functions are shown in **Table 4**. The highest biomass production was found in the atypical *M. furfur* strain at 2.84 mmol gDW^-1^ h^-1^ and the lowest was in *M. sympodialis* at 0.089 mmol gDW^-1^ h^-1^. The number of reactions that that carried flux varied among the species, ranging from 36% in the atypical *M. furfur* strain to 42% in the *M. sympodialis* strain.

**Table 4:** Predicted biomass flux with the flux balance analysis.

Strain	Biomass (mmol gDW^-1^ h^-1^)
*M. furfur*	1.280
Atypical *M. furfur*	2.840
*M. pachydermatis*	0.742
*M. globosa*	1.087
*M. sympodialis*	0.090

**FIGURE 7:**
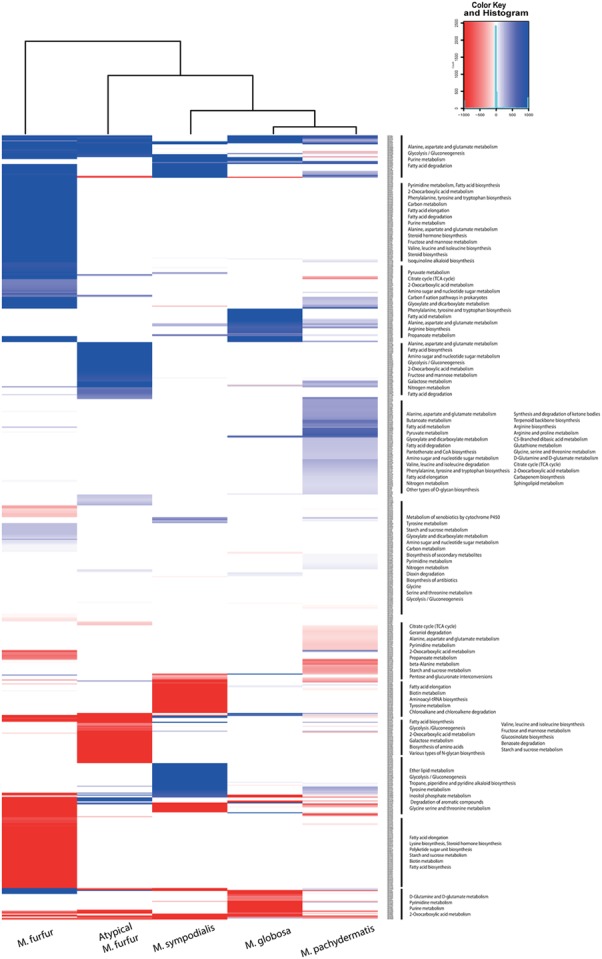
Flux distribution resulting from the flux balance analysis (FBA) of each metabolic network reaction filtered for visualization with a cutoff of ±0.05 mmol gDW^-1^ h^-1^.

The flux distribution of each network is represented in **Figure 7** for visualization as a heatmap, the resulting clusters were found based in the flux carried by each reaction, with *M. globosa* and *M. sympodialis* having the most similar flux distribution. The main differences between *M. furfur* and the other strains were reactions involved in valine, leucine, and isoleucine degradation, and pyrimidine and purine metabolism. Differences in the lipid-metabolism reactions were also found. *M. furfur* displayed high fluxes in reactions related to fatty acid degradation and elongation, which involved the conversion of hydroxyacyl-CoA to *trans*-2-enoyl-CoA. These were higher in comparison to those observed in the atypical *M. furfur* strain. Several reactions associated with fungal steroid biosynthesis were found in the atypical *M. furfur* and *M. furfur* isolates. Despite the atypical *M. furfur* isolate having a larger number of these reactions, they had higher fluxes in the *M. furfur* strain. In the atypical *M. furfur* strain, higher fluxes were found in reactions involved in the degradation of maltose, fructose, and starch. *M. globosa* and *M. sympodialis* showed similar metabolic behavior. However, some differences in *M. sympodialis* corresponding to changes in the pyruvate and gluconeogenesis pathways, as well as the pathways involved in the degradation of long-chain fatty acids into acyl-CoA, and in the conversion of hydroxyacyl-CoA to *trans*-2-enoyl-CoA were found. Finally, in *M. pachydermatis*, there were differences in the core metabolism of pyruvate and butanoate, and in the biosynthesis of phenylalanine, tyrosine, and tryptophan.

As lipid assimilation differs among *Malassezia* species, and lipid metabolism may be related to the pathogenesis of these yeasts, differences in lipid assimilation among the species was further evaluated in the model. In reactions involve in fatty acid biosynthesis such as MNXR2003 (ATP + hexadecanoic acid + CoA ⇔ AMP + palmitoyl-CoA + diphosphate), involved in the conversion of hexadecanoic acid to palmitoyl-CoA, higher fluxes were found in *M. furfur*, even though all the strains’ genomes contain the genes that codify the enzyme that catalyzes these reactions.

### Proteomic Validation

**FIGURE 8:**
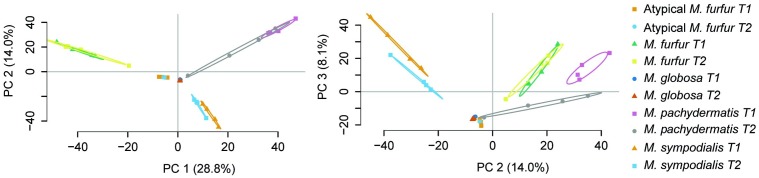
Principal component analysis (PCA) of the expressed protein cluster for the five strains at T1 (exponential phase) and T2 (stationary phase) with four replicates.

An average number of 8,864 ± 2,400 *M. furfur* peptides were found during the exponential phase (T1), corresponding to an average of 1,865 ± 154 proteins. The lowest number of peptides (828 ± 456) was obtained with *M. globosa* in the stationary phase (T2), corresponding to an average of 116 ± 60 proteins. Consensus proteins were defined as proteins present in at least two replicates with a quantitative identity of ≥95%. The number of these proteins differed among the strains. *M. furfur* in the stationary phase had the highest number (1,539 consensus proteins), while *M. globosa* presented the lowest number in the exponential phase (201 consensus proteins). The number of reactions was higher in atypical *M. furfur* in the exponential phase, with 841 reactions, as compared to the other species (**Table 5**). The principal component analysis (PCA) showed that the proteome profiles behaved differently among the species, with the samples from atypical *M. furfur* and *M. globosa* having the most similar profiles. In addition, the clustering and PCA analyses also showed that the replicates behaved similarly within each species in both the stationary and exponential phases (**Figure 8**).

**Table 5:** Proteomic profiling results of five strains of *Malassezia* during the exponential (T1) and stationary (T2) phase.

	Peptides					Proteins					Consensus		
Sample	1^∗^	2	3	4	Mean ± SD	1	2	3	4	Mean ± SD	proteins	Enzymes	Reactions
*M. furfur* (T1)	12,017	8,990	6,236	8,214	8,864 ± 2,400	2,094	1,761	1,784	1,821	1,865 ± 154	1,284	430	770
*M. furfur* (T2)	9,690	9,075	9,857	5,354	8,494 ± 2,120	2,098	1,929	2,015	1,521	1,890 ± 255	1,539	517	830
Atypical *M. furfur* (T1)	1,893	3,188	6,240	7,099	4,605 ± 2,466	864	730	1,146	1,103	960 ± 197	985	301	841
Atypical *M. furfur* (T2)	5,055	3,971	2,349	5,286	4,165 ± 1,339	930	757	582	949	804 ± 171	805	250	789
*M. pachydermatis* (T1)	7,185	9,093	7,707	8,509	8,123 ± 845	1,130	1,314	1,165	1,265	1,218 ± 85	1,118	362	716
*M. pachydermatis* (T2)	858	5,213	6,156	5,245	4,368 ± 2,380	333	844	1,130	997	826 ± 348	763	256	568
*M. sympodialis* (T1)	2,465	3,359	4,440	5,625	3,972 ± 1,366	952	1,107	1,163	1,292	1,128 ± 140	1,023	337	578
*M. sympodialis* (T2)	2,108	2,475	4,864	2,182	2,907 ± 1,314	816	918	1,234	816	946 ± 197	774	268	535
*M. globosa* (T1)	3,737	2,414	2,200	2,586	2,734 ± 686	252	247	241	224	241 ± 12	201	101	248
*M. globosa* (T2)	264	953	736	1,360	828 ± 456	71	140	63	190	116 ± 60	216	100	244

*A total of four replicates were examined for each strain. ^∗^Replicate.*

**FIGURE 9:**
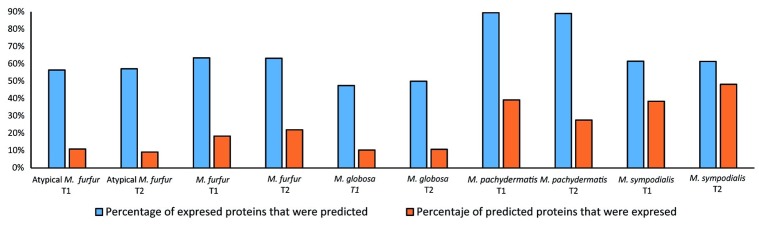
Protein validation as the percentage of enzymes predicted by the model that were expressed and the percentage of enzymes expressed that were predicted.

Protein validation of the models was conducted and expressed as the percentage of proteins predicted by the model that were detected by proteomic profiling (expressed proteins) as well as the percentage of expressed enzymes that were found to be catalyzing an reaction carrying flux in the model (**Figure 9**). Based on the validation and taking into account the percentage of the total reactions that can be experimentally validated (83% in *M. furfur*, 80% in *M. globosa*, 73% in *M. sympodialis*, 79% in *M. pachydermatis*, and 78% in Atypical *M. furfur*). The most successful model was that of *M. pachydermatis*, where ∼40 and 30% of the predicted proteins were expressed in the exponential and stationary phase, respectively. The less accurate models were those of *M. globosa* and of the atypical *M. furfur*, with ∼10% of the predicted proteins being found. Similarly, when evaluating the number of predicted enzymes that were expressed, *M. pachydermatis* showed the best prediction model with ∼90% of its expressed enzymes predicted by the flux distribution in both the exponential and stationary phases. *M. globosa*, on the contrary, only showed ∼50% of its expressed enzymes, as predicted by the model under both growth phases.

### Physiological Characterization and Requirement for Lipid Compounds for Growth

We performed a physiological characterization of these species. We used MM instead of rich media such as Sabouraud or potato dextrose agar, since these media contain small amounts of lipids that can sustain the growth of *M. pachydermatis* (Wu et al., 2015). Culturing was performed in liquid MM in the presence of oleic acid, palmitic acid, or Tween. Strains were first grown in mDixon containing a variety of lipid sources and Tween 80. To prevent subsequent growth in MM due to the presence of residual lipids either from mDixon or associated with cells, we performed a two-phase growth in MM. First, cells were diluted into MM containing oleic acid, palmitic acid, or one of the Tween sources. After 3 days, these cells were diluted again in fresh MM with oleic acid, palmitic acid, or Tween. The results are described in **Table 6** and in Supplementary Figures S4 and S5. *M. furfur* indeed can assimilate palmitic acid or oleic acid as well as all Tween variants (**Table 6** and Supplementary Figures S4, S5), including Tween 40, which represents the C16:0 fatty acid donor in this respect.

**Table 6:** Physiological characterization of *Malassezia* spp. by culturing in liquid minimal medium (MM) containing either Tw20 (Tween 20), Tw40 (Tween 40), Tw60 (Tween 60), Tw80 (Tween 80), OA (oleic acid), PA (palmitic acid), or DB (Dixon broth) during the first and second growth step.

Strain	Tw20	Tw40	Tw60	Tw80	OA	PA	DB
First growth step					
*M. furfur*	++	++	+/-	++	+/-	++	++
Atypical *M. furfur*	++	+	+	++	+/-	-	++
*M. pachydermatis*	+/-	+	++	+	+/-	+	++
*M. sympodialis*	+/-	+/-	+/-	+/-	+/-	+	++
*M. globosa*	+/-	+/-	+/-	+/-	+/-	+	++
Second growth step					
*M. furfur*	++	++	++	++	+	++	++
Atypical *M. furfur*	+	-	-	++	+	-	++
*M. pachydermatis*	-	-	-	-	-	-	++
*M. sympodialis*	-	-	-	-	-	-	++
*M. globosa*	-	-	-	-	-	-	++

*Growth was determined by measuring the absorbance of the culture at 600 nm after 168 h. -, no growth; +/-, weakly; +, good; ++, very good.*

The atypical *M. furfur* strain, however, was only able to assimilate Tween 80, Tween 20, and oleic acid. We observed that *M. pachydermatis*, *M. globosa*, and *M. sympodialis* were able to grow in the first step in MM, however, these strains were not able to grow in the second step in this defined medium supplemented with oleic acid, palmitic acid, or any of the Tween sources (**Table 6** and Supplementary Figures S4, S5). However, growth of these latter strains was sustained in the mDixon medium. Interestingly, whereas *M. furfur* was able to maintain growth in MM with palmitic acid, the atypical *M. furfur*, *M. pachydermatis*, *M. globosa*, and *M. sympodialis* growth (as determined by the CFU) declined rapidly in the second growth step (Supplementary Figure S5).

We determined whether *M. pachydermatis* and atypical *M. furfur* could sustain growth in MM with a mixture of palmitic acid and oleic acid (Supplementary Figure S6). Interestingly, we observed that both strains were then indeed able to maintain growth or survive in this mixture of saturated and unsaturated fatty acids.

## Discussion

The lipid-dependent and lipophilic life style of *Malassezia* spp. seems to involve selection pressure to adapt to skin environments rich in lipid sources in human and animal hosts. Many factors can disturb the normal status of these yeast species and lead to disease. Approaches to understanding how this disturbance occurs provide clues to managing the negative impacts on the host. Omics studies are necessary to fully comprehend these mechanisms (Jo et al., 2016). In this study, the genomes of the previously reported *M. furfur* CBS 1878 (Wu et al., 2015) and an isolate of *M. furfur* with atypical assimilation of Tween 80 were sequenced. Furthermore, the metabolic networks of five *Malassezia* strains were reconstructed via genome annotation, reaction directionality, compartmentalization, and manual curation. This allowed us to elucidate differences in the genomes, in the metabolism of fungal steroids, and other pathways among the four *Malassezia* species that were studied. Moreover, we carried out an FBA on the metabolic reconstructions to measure the potential of each strain to produce biomass. We observed differences in the flux distribution among the species, with a variation in the fluxes of reactions related to lipid metabolism. Furthermore, proteomic profiling was used to validate the results. The validation showed that most of the proteins expressed in the proteomic profiling were predicted. However, not all the model’s predictions were corroborated by this approach. Future high-throughput proteomic, metabolomic, and modeling approaches are needed to validate the models.

The genome assemblies of *M. furfur* and the atypical *M. furfur* resulted in a genome size of 14.19 and 10.3 Mb, respectively, approximately double the size of the genomes of previously sequenced species [*M. globosa* (Xu et al., 2007), *M. sympodialis* (Gioti et al., 2013), and *M. pachydermatis* (Triana et al., 2015)]. Differences in the genome size of *M. furfur* CBS 1878 (14.19 Mb) in comparison to that of the same strain reported by Wu et al. (2015) (13.5 Mb) could be due to the assembly and scaffolding methods used in each study. The total length of the resulting contigs longer than 1,000 bp in our assembly is around 13.6 Mb (**Table 1**); a similar size to the previously reported size of 13.5 Mb. Phylogenetic analysis conducted in this study established that our *M. furfur* strains are part of the *Malassezia* cluster A that has been previously described. This cluster consists of several strains of this species that were suggested to be a species complex (Wu et al., 2015). Particularly, atypical *M. furfur* (10.3 Mb) clustered with *M. furfur* 7982 (7.7 Mb), thus showing them to be distantly related to other strains, and suggesting that they are implicated in divergence events (Wu et al., 2015). More analysis is required to define the relation of the atypical strain and how it may be implicated in those events.

Additionally, the assembled genomes showed a greater number of scaffolds, indicating a possible fragmentation of the genome (de Wit et al., 2012). The fragmentation was also evidenced by the reduction in the average gene size. The fragmentation present in the genome sequence assembly may cause a bias when comparing it to the other genomes and may further generate false-positive protein annotations (Klassen and Currie, 2012; Faino et al., 2015). However, the CD-Hit and EC results provided support for our protein dataset. The CD-Hit analysis showed that the number of protein clusters was close to the number of proteins that, although higher when compared to those of the other species, did not collapse after clustering. Also, the finding of new EC numbers in the genomes, as well as EC numbers previously reported in other *Malassezia* genomes, provided support for our annotation. Additionally, the metabolic reconstruction approach allowed us to reduce the fragmentation bias by determining the possible reactions of each protein according to the domains of each gene. The genome assembly could further be improved by resequencing these two genomes with a technique that generates longer reads such as PacBio (English et al., 2012). Different mechanisms might explain the observed genome size differences. The options might include the presence of non-coding repetitions, possible protein duplications in the genomes, and/or the presence of new proteins. The first explanation could refer to mobile genetic elements such as transposons and retrotransposons as well as low-complex repetitions, as has been reported in other haploid fungi (Galagan, 2005). However, the analyses showed that less than 2% of the genomes presented these kinds of repetitions. To assess whether protein duplications or an increase in the number of unique proteins may explain the increase in the genome size of both *M. furfur* strains studied, we annotated the assemblies. The number of predicted proteins was higher in the genomes of *M. furfur* and the atypical *M. furfur* than in the genomes of *M. globosa*, *M. sympodialis*, and *M. pachydermatis*. Differences in the number of predicted proteins could not solely be explained by protein duplications, as mentioned above. The number of protein clusters detected by CD-Hit correlates with the number of total proteins. Among the number of predicted proteins found in *M. furfur* and in the atypical *M. furfur* strain (10,203 and 12,121, respectively) 7,570 and 10,434 protein clusters, respectively, were detected. Thus, although there are indeed protein duplications, most of the predicted proteins represent unique proteins in the genomes of *M. furfur* and the atypical *M. furfur* strains. This is further supported in the case of enzymes by the fact that the unique EC numbers and reactions in both *M. furfur* strains were higher than those in the other species studied. This diversification in the number of predicted proteins may provide evidence for the metabolic versatility found in *M. furfur*. The increase in unique proteins and the genetic diversification present in the *M. furfur* strains may be due to mating of the yeast, since bipolar mating systems have been previously proposed in *Malassezia* spp. (Wu et al., 2015), and sexual reproduction has been proposed to promote genetic variation in other pathogenic fungi (Ene and Bennett, 2014; Heitman et al., 2014). These special characteristics can be an advantage in terms of being able to easily adapt to different body sites, even under adverse conditions, such as blood in the case of a fungemia, which this species has been associated with as well (Barber et al., 1993). However, the analysis of metagenomic datasets from different sites on healthy human skin showed that *M. furfur* is less frequently detected than *M. globosa*, *M. restricta*, and *M. sympodialis* (Wu et al., 2015). This pattern is suggestive of the metabolic profile of this species leading to a strong and intimate relation with the host due to its complex lipid requirements.

As expected, the FAS was not found in the genomes of either *M. furfur* or the atypical *M. furfur* strains. The PKSs were found to be conserved among the *Malassezia* species, with the exception of *M. globosa*, which lacked one domain (Xu et al., 2007), and the *M. furfur* isolate, which lacked several domains in its three predicted PKSs. *Mycobacterium tuberculosis* PKSs have been associated with the biosynthesis of unique lipids or glycolipid conjugates (Quadri, 2014), and in *Streptomyces griseus*, with the synthesis of phenolic lipids (Funabashi et al., 2008). Further studies are needed to determine the importance of these unique PKSs in *Malassezia* and their role in the lipid dependency of this genus. They are clearly different from other fungi with an identity of less than 32% with the phylogenetically related basidiomycete *P. involutus* (even with this was the most similar enzyme).

Even though there were differences in the number of predicted reactions among the species, the proportion in each pathway was similar. Carbohydrate and amino acid metabolism, which are part of the core metabolism of an organism, were the most abundant, as is the case for other fungi such as *Aspergillus oryzae* (Vongsangnak et al., 2008) and *Mortierella alpine* (Ye et al., 2015). With respect to fungal steroid biosynthesis, we found that the atypical *M. furfur* strain had the highest number of reactions, followed by *M. furfur*. These reactions were almost negligible in the other three species studied. Differences in the fungal steroid biosynthesis between *M. furfur* and the other three *Malassezia* species studied may be explained by (i) the production of steroid-like fungal hormones in *M. furfur* for sexual reproduction, as happens in ascomycetes (Gooday, 1974); (ii) fungal steroids perhaps being a constitutive component of *M. furfur*, as is the case of the fruiting body of the basidiomycete *Sarcodon joedes* (Liu et al., 2013); and most likely, (iii) steroids in *M. furfur* perhaps acting as secondary metabolites, as previously reported in the basidiomycete *Polyporus ellisii* (Wang et al., 2012). Finally, the differences in arachidonic acid lipid metabolism found in the atypical *M. furfur* strain may be related to the presence of precursors of eicosanoids that may act as an alternative lipid compound in this strain (Bajpai et al., 1991). In addition, arachidonic acid may act as a mediator of skin inflammation—a previously reported role of *M. furfur* (Plotkin et al., 1998).

The topological features of the networks were used to assess their robustness. The node degree distribution (Supplementary Figures S7, S8) fits a power law regression and we showed that the five networks had free-scale topologies and non-random behavior (Barabási and Oltvai, 2004). The higher clustering and diameter of *M. sympodialis* and of the *M. furfur* strain (*M. furfur* CBS 1878) networks imply a more complex network. Yet, the clustering and diameter of these two networks were lower than for other yeast metabolic networks such as those of *S. cerevisiae* (Zomorrodi and Maranas, 2010). This may be due to the reduction in the number of connections among metabolites in each compartment when these are compartmentalized and subdivided as a representation of organelles.

The biomass fluxes found in our study were similar to those reported in other yeasts such as *S. cerevisiae* (0.7388 mmol gDW^-1^ h^-1^) (Pitkänen et al., 2014), but because of the lack of biomass data for *Malassezia* species further experimental validation of the growth rate in each species is necessary. The FBA allowed us to observe that even though the atypical *M. furfur* had a higher number of reactions for the steroid biosynthesis pathways, these had higher fluxes in the given condition in *M. furfur* strain CBS 1878. In addition, we found that *M. furfur* strain CBS 1878 displayed high fluxes in reactions related to the degradation and elongation of fatty acids such as the conversion of hydroxyacyl-CoA to trans-2-enoyl-CoA (Autio et al., 2008) than the atypical *M. furfur* strain did, and this may explain the differences in lipid assimilation observed between these two strains. Strikingly, the FBA analysis suggested an important difference in the use of palmitate. *M. furfur* had a high flux in the CoA activation of palmitate, whereas the other species lacked this activity. FASs are required in most organisms to synthesize fatty acids such as the end product palmitate (C16:0) in the cytosol and all *Malassezia* spp. lack a FAS complex and are not able to produce palmitate (Triana et al., 2015; Wu et al., 2015). This phenotype can be complemented with external lipids and Tween, which can act as donors for fatty acids, and after uptake, are directly used and/or elongated into long-chain- or very long-chain fatty acids, desaturated, or degraded via β-oxidation in the peroxisome. We observed that *M. furfur* is able to grow in MM supplemented with Tween 40 or with palmitate, indicating that palmitate is indeed used, and this is in accordance with the FBA predictions. The physiological analysis of the atypical *M. furfur* strain indicates that it cannot use palmitate since the second growth step in liquid medium supplemented with Tween 40 or with palmitate was not sustained. Similar results were obtained for *M. pachydermatis*, *M. globosa*, and *M. sympodialis* (Supplementary Figures S4, S5), which is in agreement with the FBA predictions. These findings could suggest that the transport and activation of palmitic acid in palmitic acid CoA is happening in *M. furfur*, however, could be hampered in other strains. More studies such flux variability analysis are required to clarify this. However, previous *in silico* analysis of the fatty acid metabolism of *M. globosa* using integrated microbial genomes confirmed the presence of the complete β-oxidation pathway for the degradation of saturated fatty acids (James et al., 2013). These differences could be related to differences in the fluxes in reactions related to this pathway in *M. furfur.* The observation that these latter four strains did grow in the first step in MM indicates that residual lipids from mDixon and/or associated with cells are sufficient to support the first step. These lipids are depleted in the second growth step in MM, which allows for a real analysis of lipid dependency. The observation that the atypical *M. furfur* cannot grow in MM with Tween 20, 40, and 60, the donors of C12:0, C16:0, and C18:0 fatty acids, respectively, suggests that these saturated fatty acids cannot be further elongated and/or desaturated. All strains except *M. furfur* and the atypical variant were not able to grow in MM supplemented with a single Tween or with fatty acid species, whereas they did grow in mDixon. This observation might be explained if we assume that the strains require a mixture of saturated and unsaturated fatty acids, as was similarly observed with *fasl*, *olel* (FAS and fatty acid desaturase minus) mutants of *S. cerevisiae* (Henry, 1973). Further analysis showed that the atypical *M. furfur* and *M. pachydermatis* indeed was capable to grow or survive in MM with a mixture of palmitic acid and oleic acid.

Other studies suggested that *M. globosa* and *M. restricta* are not capable of synthesizing unsaturated fatty acids due to the lack of a Δ 9-desaturase (EC 1.14.19.2) gene (Xu et al., 2007; Boekhout, 2010), which is involved in the desaturation of palmitic acid and stearic acid to palmitoleic acid and oleic acid, respectively. However, Gioti et al. (2013) found this desaturase gene in the genome of *M. sympodialis*. We also found the same gene in *M. furfur*, atypical *M. furfur*, and *M. pachydermatis*, suggesting the ability of these species to produce unsaturated fatty acids such as oleic acid, thus providing these species with an advantage in terms of their metabolic versatility to rapidly adapt to regions in which the availability of such fatty acid sources is limited. This ability is missing in *M. globosa* and *M. restricta*, which do require these unsaturated fatty acids as additional sources from their host to support their growth. These particular differences highlight the differences in the pathogenic role of these species in the development of certain dermatological diseases in which different species have epidemiological relevance, as is the case for *M. globosa* and dandruff/SD (Boekhout, 2010). In this regard, more research is required to determine how these different *Malassezia* spp. use external fatty acids.

The lipid-metabolism reactions among the other species were also divergent. Higher activity in the reactions involved in the degradation of long-chain fatty acids was present in *M. sympodialis* but not in *M. globosa* or *M. pachydermatis*. These differences may be associated with the phenotypic differences of each species, reflected by the differential lipid assimilation or the differential use of Tweens. We should, however, explore more deeply the physiology and regulation of β-oxidation in this yeast (Yarrow, 1998; Boekhout, 2010).

The higher number of reactions related to the butanoate metabolism found in *M. pachydermatis* may be related to the lipid-assimilation versatility of this species. This metabolism is closely related to the synthesis of type II and type III polyketides (which may be precursors of unique lipids) as well as to the fatty acid metabolism (Rawlings, 1998; Charrier et al., 2006). Thus, the higher activity of reactions involved in the metabolism of butanoate in *M. pachydermatis* may be reflected in the production of lipids that may not be produced by the other *Malassezia* species and that may be corroborated by lipid profiling of the yeasts.

The proteomic profiling allowed us to validate, on average, 30% of the predicted proteins of the model, implying that the other enzymes that are supposed to be expressed according to the FBA are not present or that their concentrations are substantially low. An average of 70% of the proteins expressed in all the samples were in fact predicted. Given that this technique is still not widely used to validate these kinds of models, the cutoff points and expected percentages are not well known. Furthermore, issues such as the identification of large-scale protein datasets, small protein concentration detection, and the extraction of low soluble proteins such as membrane proteins may affect the profiling (Marcotte, 2007; Yates, 2013).

Together, the metabolic reconstruction and modeling showed versatility within the genus of *Malassezia*. Flux differences were suggested in the production of steroids in *M. furfur* and in butanoate metabolism in *M. pachydermatis*. The assimilation defects of palmitic acid were suggested in *M. globosa*, *M. sympodialis*, *M. pachydermatis*, and in the atypical variant of *M. furfur*. The capability of *M. furfur* to assimilate palmitic acid was confirmed via culturing on defined media.

## Author Contributions

ST, AC, AG, HdC, and SR contributed to the design of the work. ST, AC, and RO performed the experiments. All authors were involved in the analysis and interpretation of data. ST and AC wrote the manuscript. HdC, HW, SR, and AG made revisions. All authors approved the version to be published and agreed to be accountable for all aspects of the work.

## Conflict of Interest Statement

The authors declare that the research was conducted in the absence of any commercial or financial relationships that could be construed as a potential conflict of interest. The reviewer LK and handling Editor declared their shared affiliation.

## Footnotes

1 http://repeatmasker.org

2 http://hmmer.org

3 http://proteomics.ucdavis.edu/
