# Supplementary material for: Lipid Metabolic Versatility in Malassezia spp. Yeasts Studied through Metabolic Modeling
Source: Front Microbiol. 2017 Sep 14;8:1772. doi: 10.3389/fmicb.2017.01772 (PMC5603697; doi:10.3389/fmicb.2017.01772)
Supplement: Supplementary file 1 [file Data_Sheet_1.DOCX]

**Genome-scale metabolic reconstructions of *Malassezia* spp. unveils new insights in their lipid metabolism**

Sergio Triana^1,2^, Hans de Cock^3^, Robin A. Ohm, Giovanna Danies^1^, Han A. B. Wösten^3^, Silvia Restrepo^1^, Andrés Fernando Gonzalez Barrios,^2^* and Adriana Celis^1,3^*

^1^ Department of Biological Sciences, Universidad de los Andes, Bogotá, Colombia

^2^ Grupo de Diseño de Productos y Procesos (GDPP) Chemical Engineering Department, Universidad de los Andes, Bogotá, Colombia

^3^ Microbiology, Department of Biology, Faculty of Science, Utrecht University, Utrecht, the Netherlands

***Corresponding author**

Mailing addresses: [andgonza@uniandes.edu.co](mailto:andgonza@uniandes.edu.co), [acelis@uniandes.edu.co](mailto:acelis@uniandes.edu.co)

# Supplementary Data

**File S1.** Metabolic network reconstructions of *Malassezia globosa, Malassezia sympodialis, Malassezia pachydermatis, Malassezia furfur,* and atypical *Malassezia furfur.*

## Supplementary Tables

**Table. S1** Biomass composition used as an objective function.

| **KEGG ID** | **Compound** | **Coeﬃcient** | **METANETX ID** |
| --- | --- | --- | --- |
| C00001 | -59,276 | H2O | MNXM2 |
| C00002 | -59,276 | atp | MNXM3 |
| C00965 | -11,348 | 1,3-beta-D-Glucan | MNXM6492 |
| C00096 | -0.8079 | GDP-mannose | MNXM82 |
| C00369 | -0.5185 | starch/glycogen | MNXM93732 |
| C00041 | -0.4588 | ala-L | MNXM32 |
| C00025 | -0.3018 | glu-L | MNXM89557 |
| C00049 | -0.2975 | asp-L | MNXM42 |
| C00123 | -0.2964 | leu-L | MNXM140 |
| C00037 | -0.2904 | gly | MNXM29 |
| C00047 | -0.2862 | lys-L | MNXM78 |
| C00183 | -0.2646 | val-L | MNXM199 |
| C00407 | -0.1927 | ile-L | MNXM231 |
| C00188 | -0.1914 | thr-L | MNXM142 |
| C00065 | -0.1854 | ser-L | MNXM53 |
| C00148 | -0.1647 | pro-L | MNXM114 |
| C00062 | -0.1607 | arg-L | MNXM70 |
| C00079 | -0.1339 | phe-L | MNXM97 |
| C00064 | -0.1054 | gln-L | MNXM37 |
| C00082 | -0.102 | tyr-L | MNXM76 |
| C00152 | -0.1017 | asn-L | MNXM147 |
| C00135 | -0.0663 | his-L | MNXM134 |
| C00105 | -0.0599 | ump | MNXM80 |
| C00073 | -0.0507 | met-L | MNXM61 |
| C00144 | -0.046 | amp | MNXM113 |
| C00020 | -0.046 | gmp | MNXM14 |
| C00055 | -0.0447 | cmp | MNXM31 |
| C00078 | -0.0284 | trp-L | MNXM94 |
| C01083 | -0.0234 | tre | MNXM198 |
| C00059 | -0.02 | SO4 | MNXM58 |
| C00097 | -0.0066 | cys-L | MNXM55 |
| C00364 | -0.0036 | damp | MNXM257 |
| C00360 | -0.0036 | dtmp | MNXM432 |
| C00239 | -0.0024 | dcmp | MNXM266 |
| C00362 | -0.0024 | dgmp | MNXM546 |
| C05437 | -0.0015 | zymst | MNXM574 |
| C00255 | -0.00099 | rib | MNXM270 |
| C01694 | -0.0007 | ergst | MNXM922 |
| C00010 | -0.000001 | NAD | MNXM12 |
| C00575 | -0.000001 | CoA | MNXM243 |
| C00461 | -0.000001 | FAD | MNXM46301 |
| C00051 | -0.000001 | gthrd | MNXM57 |
| C00101 | -0.000001 | thf | MNXM79 |
| C00003 | -0.000001 | chitin | MNXM8 |
| C00016 | -0.000001 | camp | MNXM96415 |
| C00035 | 0.8079 | GDP | MNXM30 |
| C00008 | 59,276 | ADP | MNXM7 |
| C00009 | 59,305 | Orthophosphate | MNXM9 |
| C00080 | 11,740,002 | H | MNXM1 |

## Supplementary Figures

**Figure S1**. Dot plot of the Nucmer pairwise genome alignments.

**Figure S2**. Dot plot of the Promer pairwise genome alignments.

**Figure S3***.* A. Distribution of reactions from the core metabolism of the five *Malassezia* strains. B. In red, the pathways from KEGG present in the core metabolism of the five *Malassezia* strains**.**

**Figure S4.** Physiological characterization of *Malassezia* spp. used in this study via culturing in liquid minimal medium (MM) containing either Tw20 (Tween 20), Tw40 (Tween 40), Tw60 (Tween 60), Tw80 (Tween 80), OA (oleic acid), PA (palmitic acid), or DB (Dixon Broth) during the first (A) and second growth step (B). Measurements are at OD 600 nm.

**Figure S5.** Growth curve of *Malassezia* species used in this study via culturing in liquid minimal medium (MM) containing palmitic acid (4 mM).

**Figure S6.** Growth curve of *M. furfur* CBS 1878 and atypical *M. furfur* (4DS) and *M. pachydermatis* in liquid minimal medium (MM) containing palmitic acid and oleic acid (4 mM)

**Figure S7***.* In-degree distribution of the node from each metabolic network of *Malassezia globosa* (A)*, Malassezia sympodialis* (B)*, Malassezia pachydermatis* (C)*, Malassezia furfur* (D)*,* and atypical *Malassezia furfur* (E)*.*

**

**Figure S8.** Out-degree distribution of the node from each metabolic network of *Malassezia globosa* (A)*, Malassezia sympodialis* (B)*, Malassezia pachydermatis* (C)*, Malassezia furfur* (D)*,* and atypical *Malassezia furfur* (E)*.*
